# Mean Serum Creatine Kinase among Organophosphate Poisoning Cases in a Tertiary Care Centre: A Descriptive Cross-sectional Study

**DOI:** 10.31729/jnma.7692

**Published:** 2022-10-31

**Authors:** Saru Twayana, Vijay Kumar Sharma, Mithileshwor Raut, Aseem Bhattarai, Binod Kumar Yadav, Sangha Ratna Bajracharya, Eans Tara Tuladhar

**Affiliations:** 1 Department of Biochemistry, Maharajgunj Medical Campus, Maharajgunj, Kathmandu, Nepal; 2 Department of Pharmacology, Maharajgunj Medical Campus, Maharajgunj, Kathmandu, Nepal

**Keywords:** *acetylcholinesterase*, *creatine kinase*, *organophosphate poisoning*, *rhabdomyolysis*

## Abstract

**Introduction:**

Major cases of poisoning are associated with organophosphates. Cholinergic effects and an intermediate phase seen with organophosphate poisoning may implicate myopathy. Creatine kinase is a marker of muscle tissue damage. This study aimed to find out the mean serum creatine kinase among organophosphate poisoning cases in a tertiary care centre.

**Methods:**

A descriptive cross-sectional study was carried out among organophosphate poisoning cases in a tertiary care hospital from 13 October 2017 to 30 March 2018. Ethical approval was taken from the Institutional Review Committee [Reference number: 117(6-11-E) 2/074/075]. Blood samples were assayed for serum acetylcholinesterase in the pharmacology laboratory and for serum creatine kinase and lactate dehydrogenase in the biochemistry laboratory. Low serum acetylcholinesterase was taken as the basis for the establishment of organophosphate poisoning. A convenience sampling technique was used. Point estimate and 95% Confidence Interval were calculated.

**Results:**

Among 103 organophosphate poisoning cases, the mean serum creatine kinase was 931.35±446.60 IU/l (845.10-1017.60, 95% Confidence Interval).

**Conclusions:**

The mean serum creatine kinase level among organophosphate poisoning cases was higher than in other studies done in similar settings.

## INTRODUCTION

Major cases of poisoning in Nepal are associated with organophosphatesth rough suicidal intention,accidental consumption, or occupational hazards.^12^ One of the toxic effects of organophosphorus compounds is the over-activation of cholinergic receptors at the neuromuscular junctions and intermediate phaseleading to muscle injury or myopathy.^3^

Creatine kinase (CK) is used in diagnosing myopathy as markers of tissue damage. CK is an intramuscular enzyme facilitating skeletal and cardiac muscle to meet variable energy demands.^4^ The excess excitement of skeletal muscle in organophosphate (OP) poisoning may lead to myopathy or muscle injury with the release of CK into the blood.^3,4^

This study aimed to find out the mean serum creatine kinase among organophosphate poisoning cases in a tertiary care centre.

## METHODS

A descriptive cross-sectional study was conducted in the Department of Biochemistry of Tribhuvan University Teaching Hospital (TUTH) from 14 October 2017 to 30 March 2018. Ethical approval was obtained from the Institutional Review Committee of the same institute [Reference number: 117(6-11-E)^2^/074/075]. The serum samples of patients with low serum cholinesterase levels as reported in the pharmacology laboratory of TUTH with a history of OP poisoning during the study period were included in the study. Patients with a history of liver disease and renal disease were excluded. The convenience sampling method was used. The sample size was calculated using the following formula:


$$\begin{array}{ll} \text{n} & ={\text{Z}}^{2}\times \frac{\sigma^{2}}{{\text{e}}^{\text{2}}}\\ & =1.96^{2}\times \frac{77.89^{2}}{15^{2}}\\ & =104 \end{array}$$

Where,

n= minimum required sample size
Z= 1.96 at 95% Confidence Interval (CI)
σ = standard deviation of serum creatine kinase, 77.89^5^
e= margin of error, 15%

The calculated minimum sample size was 92. However, 103 OP poisoning cases were included in this study. Serum cholinesterase was analysed using the commercially available kit on the market (BIOLABS, France) which was based on the cholinesterase butyryl thiocholine method. The assay-based range from the BIOLABO kit was taken as the cut-off value (Male: 5900-12200 IU/l, Female: 4700-10400 IU/l). Acetylcholinesterase below this range was taken as confirmed cases of OP poisoning.^6^ These same serum samples were then analysed for CK in the biochemistry laboratory of TUTH. The enzymes were estimated using the commercially available analysis kit (BIOLABO, France) which was based on the kinetic IFCC (International Federation of Clinical Chemistry) method for CK. The analysis was carried out in autoanalyzer BT1500. The sample was stored at 4°C till analysis. Analysis was carried out within 24 hours of obtaining the sample. Creatine kinase below 200 IU/ml for males and 180 IU/l for females was taken as the normal range defined by the analysis kit.

Data were entered and analysed by IBM SPSS Statistics 20.0. Point estimate and 95% CI were calculated.

## RESULTS

**Figure 1:**
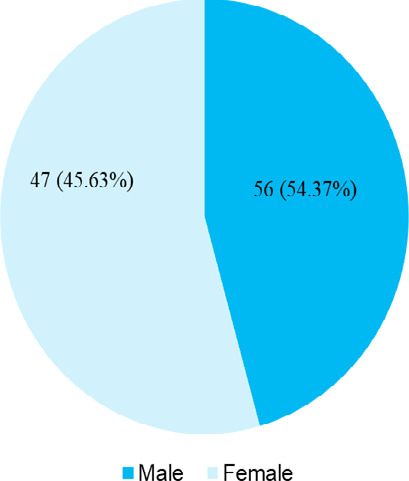
Gender-wise distribution (n= 103).

Among 103 organophosphate poisoning cases, the mean serum creatine kinase was 931.35±446.60 IU/l (845.10-1017.60, 95% CI). There were 47 (45.63%) males and 56 (54.37%) females (Figure 1).

All the 47 (100%) males showed CK levels higher than 200 IU/l whereas only 50 (89.30%) females showed CK levels more than 180 IU/l (Table 1).

**Table 1:** Mean creatine kinase levels according to gender (n= 103).

Parameters	Creatine kinase (IU/l) Mean±SD (Range)
Male (n= 47)	1108.23±432.30 (223-1659)
Female (n= 56)	782.91±405.48 (68-1360)
Total (n= 103)	931.35±446.60 (68-1659)

A total of 97 (94.17%) cases had high CK. The mean age of total, male and female cases was 29.56±13.18, 33.15±15.44, and 26.55±10.13 years. The median serum acetylcholinesterase among organophosphate poisoning cases was 874 IU/l (Table 2).

**Table 2:** Serum acetylcholinesterase level (n= 103).

Parameters	Acetylcholinesterase(IU/l) Median (Q1, Q3)
Males (n= 47)	725 (566, 243)
Females (n= 56)	1388 (555.50, 5733.25)
Total (n= 103)	874 (566, 5570)

## DISCUSSION

Our study constituted 103 organophosphate poisoning cases that had their acetylcholine esterase level below the reference range. Even though a smaller proportion of 10.52% of females had their serum CK levels in the normal range, all males enrolled in this study had CK levels higher than the reference range. As creatine kinase is a marker of muscle injury, we can claim that organophosphate poisoning is associated with muscle injury or myopathy.^3,4^

Excessive cholinergic along with intermediate phase can lead to muscle injury.^3^ Biochemical parameters (such as serum CK) were found to predict the development of respiratory paralysis.^7^ Predominant cholinergic effects are seen when serum cholinesterase is less than 50% normal range.^8^ Few studies have reported a significant positive correlation between CK with the severity of organophosphate poisoning.^5,7,9^ This might be the reason that we found raised serum creatine kinase levels in a higher proportion of cases (94%) in contrast to the studies done in India (24%),^10^ and (8%).^11^ The mean age of our study population (29.56 years) is found to be similar to other studies done in India (25.5 years) and (33.23 years).^5,10^

Males suffering from organophosphate poisoning had lower serum acetylcholinesterase levels (725 IU/l vs 1388 IU/l ) and higher serum CK levels (1108.23 IU/l vs 782.91 IU/l) than females. However, we also need to consider that males have more muscle mass than females. Mean serum CK level in males was as elevated as seen in the intermediate phase but the patients were not followed up.^12^ Our study has shown that females outnumbered males in organophosphate poisoning as with other studies carried out in Nepal,^2,8^ in contrast to the studies done in India which have shown male preponderance.^11,12^

Another tissue injury marker, LDH has also been reported to be elevated in organophosphate poisoning in many studies.^7,13^ Creatine kinase (81 kDa) being of lower molecular weight than lactate dehydrogenase (144 kDa) may be the reason for its spillage into the blood faster, thus the early indicator of tissue damage.^14^

This study is based on laboratory findings in diagnosed cases of organophosphorus poisoning. A description of the clinical profile of patients is lacking in this study. Further studies with the clinical profile need to be considered.

## CONCLUSIONS

The serum creatine kinase level in organophosphate poisoning cases was higher than the standard data comparable to the studies done in similar settings.
